# Quality Information Detection of *Agaricus bisporus* Based on a Portable Spectrum Acquisition Device

**DOI:** 10.3390/foods12132562

**Published:** 2023-06-30

**Authors:** Jiangtao Ji, Yongkang He, Kaixuan Zhao, Mengke Zhang, Mengsong Li, Hongzhen Li

**Affiliations:** 1 College of Agricultural Equipment Engineering, Henan University of Science and Technology, Luoyang 471003, China; jjt0907@163.com (J.J.); ukon.he@gmail.com (Y.H.); mkzhang0328@163.com (M.Z.); lhz13014731023@163.com (H.L.); 2 Collaborative Innovation Center of Machinery Equipment Advanced Manufacturing of Henan Province, Henan University of Science and Technology, Luoyang 471003, China; 3 Science & Technology Innovation Center for Completed Set Equipment, Longmen Laboratory, Luoyang 471023, China

**Keywords:** *Agaricus bisporus*, spectrum analysis, moisture content, whiteness measurement

## Abstract

As one of the most popular edible fungi in the market, the quality of *Agaricus bisporus* will determine its sales volume. Therefore, to achieve rapid and nondestructive testing of the quality of *Agaricus bisporus*, this study first built a portable spectrum acquisition device for *Agaricus bisporus*. The Ocean Spectromeper was used to calibrate the spectral data of the device, and the linear regression analysis method was combined to analyze the two. The results showed that the Pearson correlation coefficient of significance between the two was 0.98. Then, the spectral data of *Agaricus bisporus* were collected, the spectral characteristic wavelength of *Agaricus bisporus* was extracted by the SPA and PCA algorithms, and the moisture content and whiteness prediction models based on a BP neural network and PLSR, respectively, were built. The parameters of the BP neural network model were optimized by SSA. The R^2^ values for the final moisture content and the predicted whiteness were 0.95 and 0.99, and the RMSE values were 5.04% and 0.60, respectively. The results show that the portable spectral acquisition and analysis device can be used for the accurate and rapid quality detection of *Agaricus bisporus*.

## 1. Introduction

*Agaricus bisporus* (white mushroom) is a common edible fungus in the food industry and widely exists in the edible fungus industry in various countries. Because of its white color, delicious taste and high nutritional value, it is widely welcomed by consumers [1,2]. The external quality of *Agaricus bisporus* largely determines the level of consumers’ desire to buy it. Most of the existing quality evaluation methods of *Agaricus bisporus* are mainly based on appearance evaluation, which is easily affected by subjective factors [3,4]. The traditional manual detection method is time-consuming, laborious and poor in timeliness. To improve the production efficiency and sales of *Agaricus bisporus*, a rapid, nondestructive and portable method for the quality determination of *Agaricus bisporus* is urgently needed.

Visible/near infrared spectroscopy is an accurate, rapid and nondestructive method for the analysis of chemical compounds. In recent years, spectral detection technology has been widely used in crop quality, fruit quality, food quality and safety detection [5,6,7,8,9,10]. To date, some scholars have used spectral detection technology for chemical composition analysis and quality detection of edible fungi. For example, Mollazade et al. [11] collected the spectral information of bisporus samples based on hyperspectral imaging technology; eight key wavelengths reflecting the browning of *Agaricus bisporus* were detected by a competitive adaptive reweighted sampling (CARS) algorithm, and partial least squares discriminant analysis (PLS-DA) was used as a calibration model. The separation of different degrees of *Agaricus bisporus* browning development was obtained, and the browning development degree of *Agaricus bisporus* can be accurately detected. Lin et al. [12] used reflection mode near-infrared hyperspectral imaging to quickly and losslessly predict and visualize the water content (MC) in *Agaricus bisporus* slices, and the moisture distribution of *Agaricus bisporus* slices and whole slices during microwave-vacuum drying was visualized by combining spectral data preprocessing and modeling methods. The prediction accuracy of the final model for the moisture content of *Agaricus bisporus* slices and whole slices reached 98.5% and 96.3%, respectively. Baskar et al. [13] proposed an analytical technique using attenuated total reflection-Fourier transform infrared spectroscopy (ATR-FTIR) as a nondestructive testing tool to evaluate the moisture content of bisporus samples, which provided a method for the rapid determination of the storage life of *Agaricus bisporus* in the edible *Agaricus bisporus* industry. Parrag et al. [14] used hyperspectral images to test the quality of mushroom bisporus and successfully separated the infected samples of cobweb disease by using the support vector machine method. The proportion of correct classification in each group of samples was more than 80%, and the proportion of correct classification in the verification process was more than 75%. Xiao et al. [15] demonstrated a rapid, nondestructive, and accurate near-infrared hyperspectral imaging (NIR-HSI) technique for the visualization and prediction of soluble solids content (SSC) of *Agaricus bisporus* during ingestion.

With the miniaturization of detection equipment, portable devices have also been developed in the field of nondestructive detection of agricultural products [16,17,18,19]. Based on the principle of visible/near infrared local reflection, a portable spectrum acquisition device was built according to the shape characteristics of *Agaricus bisporus* to realize the rapid and nondestructive detection of the its moisture content and whiteness of *Agaricus bisporus*, which provides technical support for promoting the development of the *Agaricus bisporus* industry.

## 2. Materials and Methods

### 2.1. Agaricus bisporus Sample Preparation

The *Agaricus bisporus* samples needed in the experiment were purchased from Luoyang Aojite Agricultural Development Co., Ltd. (Luoyang, China), and were sent to the laboratory after being sorted by professional sorting operators in the factory. In this study, a total of 360 fresh A. bisporus mushrooms with similar size and no obvious damage on the surface were used, including 200 for moisture content and 160 for whiteness detection. Spectral acquisition on all mushrooms was performed. After the spectral acquisition, 200 of the mushrooms were dried to measure their moisture content, and another 160 mushrooms were graded on whiteness according to the standard in Table 1.

The moisture content of *Agaricus bisporus* was determined by the direct drying method (also known as the oven-drying method) according to the determination standard of edible fungi GB/T 5009.3-2010. A part of the *Agaricus bisporus* detection part was cut out, and its fresh weight was weighed as m_1_ with a precision electronic balance (m_1_ is between 4 and 7 g, accurate to 0.001 g). The precision electronics used are WT1003 produced by Hangzhou Wante Weighing Instrument Co., Ltd. (Hangzhou, China), with a measuring range of 100 g, an index value of 0.001 g, a weighing pan diameter of 100 mm, and an external size of 200 × 240 × 140 mm. After weighing, the sample was placed into a clean aluminum tray, transferred into an electric blast drying oven at 105 °C, and dried for 2 h. After reaching the drying time, the sample was cooled in the drying oven and weighed, and then, the drying was repeated until the weight difference between the two times did not exceed 0.002 g. The dry weight at this point was recorded as m_2_. The true value of the whiteness of *Agaricus bisporus* was obtained by placing the *Agaricus bisporus* sample in a standard light source box (D65 standard light source) for sensory evaluation and grading according to the artificial grading standard of *Agaricus bisporus*. The grading standard is shown in Table 1. Forty samples of each grade of *Agaricus bisporus* were selected, and four groups of diffuse reflectance spectral data of *Agaricus bisporus* were collected by the Ocean Spectromeper and a self-developed device. Then, the whiteness value was calculated.

### 2.2. Portable Agaricus bisporus Spectrum Acquisition Device

**Figure 1:**
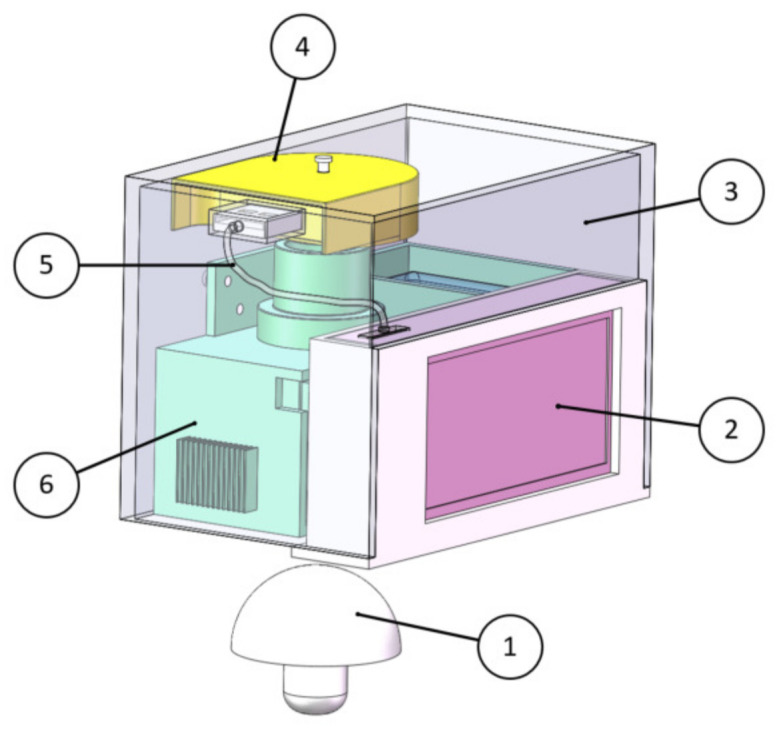
Three-dimensional (3D) image of portable *Agaricus bisporus* spectrum acquisition device. 1. *Agaricus bisporus*; 2. Spectrum processing unit; 3. Shell; 4. Spectrum acquisition module; 5. Data line; 6. Light source module.

In this work, a portable spectrum acquisition device for *Agaricus bisporus* was built based on the spectral information technology, optical knowledge and physiological structure characteristics of *Agaricus bisporus*, which was mainly composed of a light source module, spectrum acquisition module, spectrum processing unit, shell and so on. The 3D image of the portable *Agaricus bisporus* spectrum acquisition device is shown in Figure 1.

**Figure 2:**
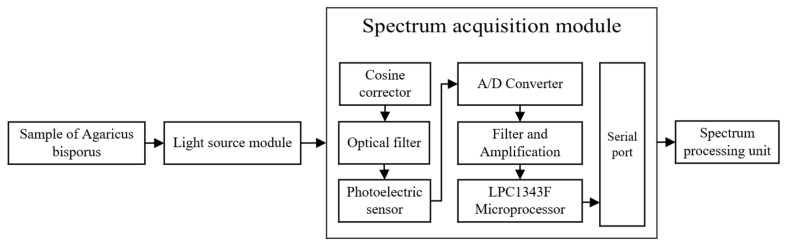
Schematic diagram of the portable spectrum acquisition device for *Agaricus bisporus*.

A schematic diagram of the portable *Agaricus bisporus* spectrum acquisition device is shown in Figure 2. A light source emits diffuse reflection light to irradiate the surface of the *Agaricus bisporus*. Light that enters the interior of the *Agaricus bisporus* and is reflected back to carry an *Agaricus bisporus* spectrum passes through a cosine calibrator to obtain parallel light. The parallel light passes through an optical filter to obtain a spectrum in a visible light range, the spectrum is received by a photoelectric sensor, and the obtained spectrum data are subjected to A/D conversion. The spectrum data processed by the LPC1343F microprocessor after filtering and amplifying processing are transmitted to the spectrum processing unit, the spectrum processing unit further processes and studies the obtained spectrum data of *Agaricus bisporus*, and the processing result is displayed on a displayer.

#### 2.2.1. Spectrum Acquisition Module

**Figure 3:**
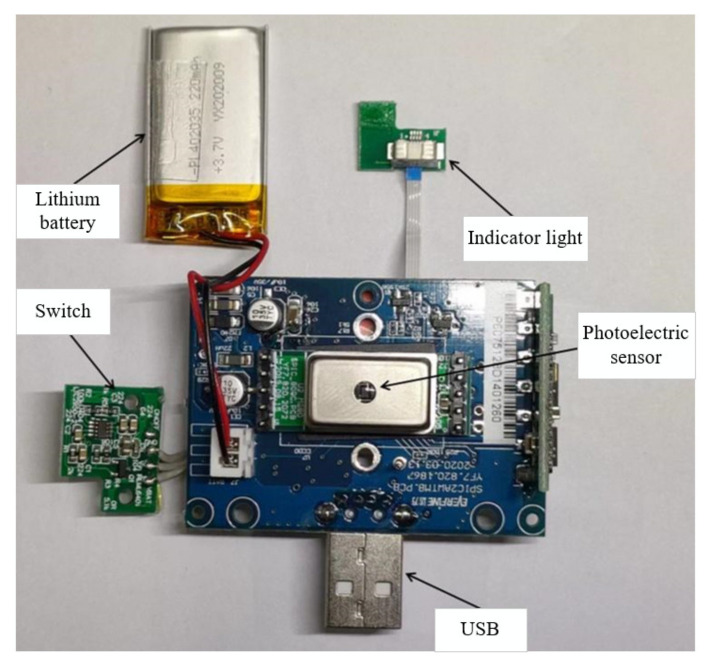
The physical image inside the spectrum acquisition module.

The spectrum acquisition module takes a silicon photocell as the core of the acquisition module and is used to receive the diffuse reflection optical information on the surface of the *Agaricus bisporus*, converting a received optical signal into an electric signal into optical spectrum data, and finally transmitting the acquired optical spectrum data to an optical spectrum processing unit through a serial port to realize the acquisition of spectral data of *Bisporus bisporus*. The receiving wavelength range is 400–800 nm. The physical image inside the spectrum acquisition module is shown in Figure 3. 

#### 2.2.2. Light Source Module

**Figure 4:**
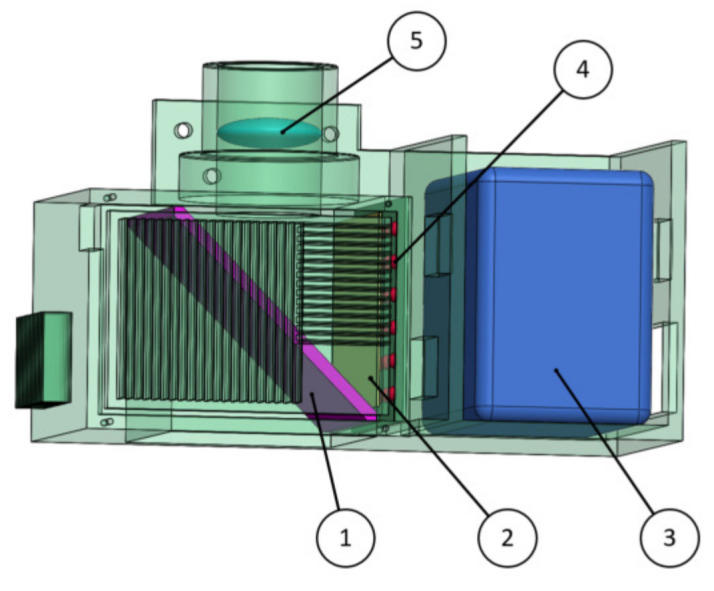
Three-dimensional (3D) drawing of the light source module. 1. Semilens surface; 2. Diffusion plate; 3. Power supply; 4. LED lamp bead; 5. Lens.

The internal 3D structure of the light source module is shown in Figure 4, which is mainly composed of a power supply, LED lamp bead, diffusion plate, lens and semilens surface. The LED lamp bead color temperature is 6500 K, and its optical performance is very close to that of the D65 standard light source.

**Figure 5:**
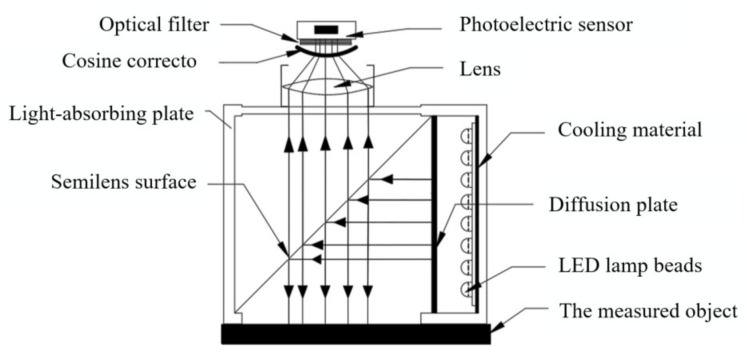
Optical path image.

A diffusely reflected light source path is shown in Figure 5. The light rays emitted by the LED lamp beads are irradiated on a 45-degree semilens surface through the diffusion plate to form uniform diffusion light, and when the *Agaricus bisporus* receives the vertical diffusion light, the diffusion light is reflected to the upper lens to form concentrated light spots through the lens, and the concentrated light spots are finally received by the spectrum acquisition module.

#### 2.2.3. Device Integration

**Figure 6:**
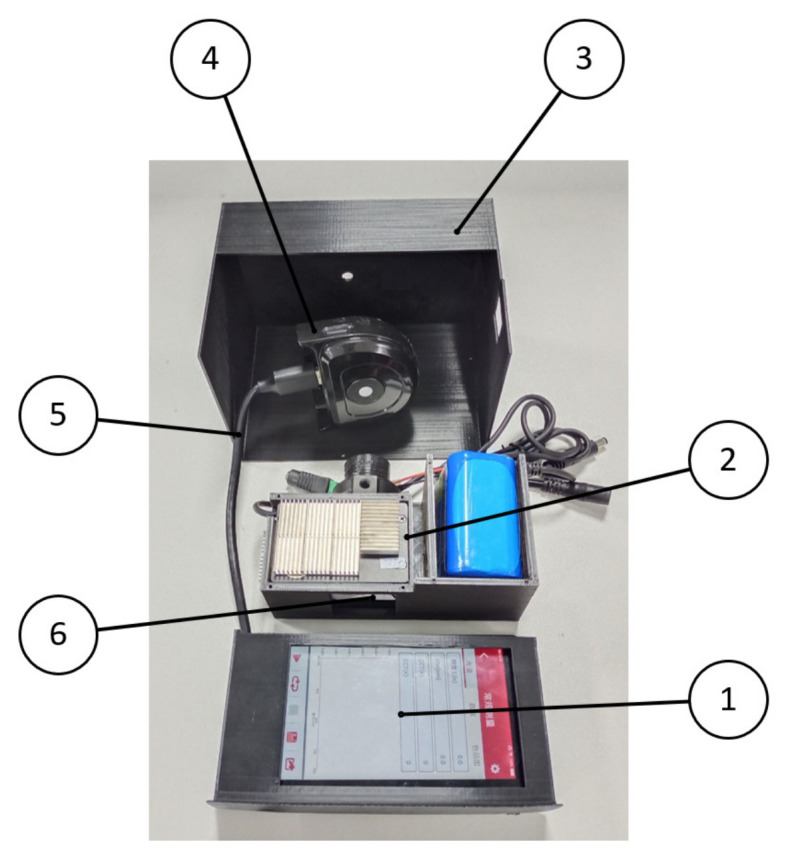
Physical image of portable *Agaricus bisporus* spectrum acquisition device. 1. Spectrum processing unit; 2. Light source module; 3. Shell; 4. Spectrum acquisition module; 5. USB cable; 6. Acquisition window.

**Figure 7:**
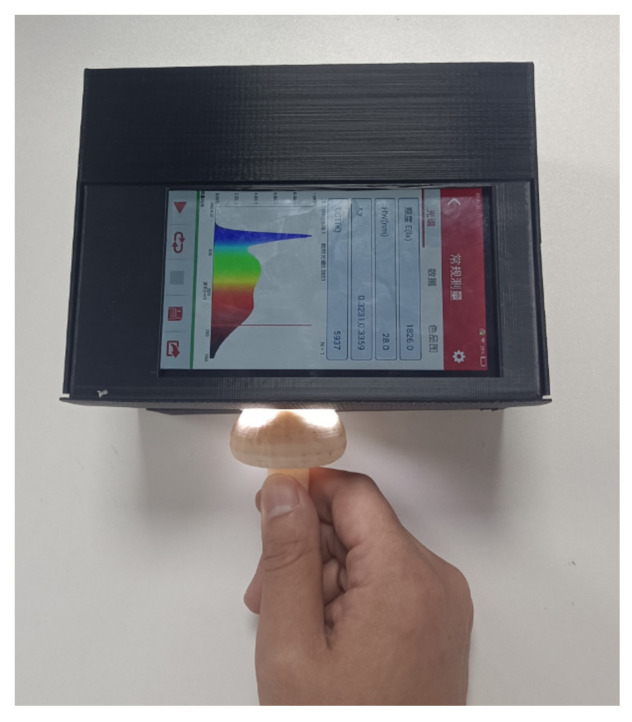
Portable Spectrum Acquisition Device for *Agaricus bisporus*.

The physical image of the portable spectrum acquisition device for *Agaricus bisporus* built in this work is shown in Figure 6, and the device’s overall size is 150 mm × 110 mm × 100 mm. Considering the influence of noise and other interference factors, the spectral acquisition range of 450–760 nm data is taken, and the integration time is adaptive. The measurement of the portable spectrum acquisition device for *Agaricus bisporus* is shown in Figure 7.

The spectrum acquisition device is a microcomputer based on the Android operating system, and it mainly includes two parts (data processing and display) and can complete the functions of spectral data preprocessing, data analysis, display and storage. The display screen is used to display the spectral data received by the spectrum acquisition device and the result of data processing. The core of the data processing module is the microprocessor, which is used to preprocess the spectral data obtained from the spectrum acquisition module. Then, the preprocessed data are written into the algorithm model to obtain the prediction results, which are displayed on the display screen.

### 2.3. Spectrum Acquisition, Calibration and Preprocessing

To calibrate the error caused by the change in external factors in the detection process of the spectrum acquisition device, it is necessary to calibrate and preprocess the spectrum data collected by the spectrum acquisition device. In this article, the calibration was carried out using a reference spectrometer, which is a product manufactured by Ocean Optics, model USB4000. The effective spectral range of this spectrometer is 200–1100 nm, the sensitivity is 130 photons/count (400 µm), the optical resolution is 0.3~10.0 nm FWHM, there are a total of 3648 bands, and the overall dimensions are 81.9 mm × 63.3 mm × 34.4 mm.

#### 2.3.1. Spectrum Acquisition

The spectral data were collected using the Ocean spectrometer and the portable spectrometer we constructed, respectively. When using the ocean optical spectrometer, the fiber probe was placed about 15 mm above the mushrooms. When using the portable spectral acquisition equipment, the mushroom was kept close to the acquisition window to avoid ambient light entry. The collection was performed by hand holding, as shown in Figure 7. The spectral acquisition device was calibrated and whiteboard calibrated before data acquisition, and dark signal correction was performed every 20 samples during the detection process. The spectrometer and light source we built are in an almost pure black 3D printed shell; therefore, the calibration is carried out under the condition of closing the detection window of this device. The test is carried out under indoor natural light conditions. The spectrum acquisition device is placed on the upper end of the test sample. To avoid external light interference as much as possible, the sample is placed under a black background and closely attached to the light outlet of the light source. The diffuse reflection method is used for measurement. The wavelength of the spectrum is automatically collected at an interval of 1 nm, and the sample is attached to the center of the light outlet of the light source when the spectrum is collected. The measurement was repeated 3 times for each sample, and the average value of the collected radiation flux was taken as the radiation flux of the sample.

#### 2.3.2. Spectral Calibration 

To verify the accuracy of this research device, the Ocean Spectrometer was used as the standard control group in this experiment, and a spectral acquisition test was carried out on *Agaricus bisporus*. The spectral data were calibrated according to the following Formulas (1)–(3):

(1) $$\Delta P=P_{2}-P_{1}$$

(2) $$\bar{P}=\frac{\sum_{i=1}^{N}\Delta P_{i}}{N}$$

(3) $$P\prime =P_{2}+\bar{P}$$

where *P*_1_ refers to the data collected by the Ocean spectrometer, *P*_2_ refers to the data collected by the apparatus of this study, Δ*P* refers to the difference between *P*_1_ and *P*_2_ corresponding to each sample, $\bar{P}$ refers to the mean of all samples Δ*P*, and *P*′ refers to the calibrated spectral data.

#### 2.3.3. Spectral Preprocessing

In the process of spectral acquisition, due to the influence of ambient light and power stability, there will be different degrees of noise interference in the collected spectral data. Before the quantitative calculation of the spectral data, the spectral data are preprocessed to filter out the redundant information in the spectrum to the maximum extent and improve the accuracy of the model. In this work, four noise reduction methods, namely, Savitzky–Golay (SG), normalization, standard normalized variate (SNV) and multiplicative scatter correction (MSC), were used to process the spectral data of *Agaricus bisporus* [20,21,22,23].

### 2.4. Dimensionality Reduction Method

#### 2.4.1. Successive Projections Algorithm

The successive projection algorithm (SPA) [24] is a forward circular selection method that uses the vector projection analysis method to select the effective wavelength with the minimum redundancy and collinearity. In this method, a new variable is added in each iteration after the first cycle until the wavelength with the largest projection vector is obtained, which is used as the candidate wavelength, and finally, the characteristic wavelength combination is selected according to the model.

#### 2.4.2. Principal Component Analysis

Principal component analysis (PCA) [25] is one of the most widely used mathematical dimensionality reduction methods, and it aims to project high-dimensional data to low-dimensional space according to the linear projection method, expecting to obtain as much information as possible to reduce the data dimension and retain more features of the original data points.

### 2.5. Moisture Content Detection Method

#### 2.5.1. BP Neural Network

In this experiment, the standard value of the *Agaricus bisporus* moisture content was measured by an electric blast drying oven. The drying time was first set to constant weight, and the moisture content was finally calculated by the moisture content formula. The neural network used is a three-layer network structure built using MATLAB, including an input layer, a hidden layer, and an output layer. The tansig function and purelin function are used for the activation function of the implicit layer and the output layer, respectively, and the trainlm function is used for the training function.

The number of input nodes of the BP model [26,27] is determined by the dimension of the input data. To reduce the size of the model, we used SPA and PCA to reduce the dimensions of the input data. The middle layer usually has 5–10 nodes. We compared the results for each model at different numbers of nodes in the middle layer, and we selected the number of nodes with the highest accuracy. To ensure the prediction accuracy of the BP neural network model, the training set and the test set of the BP neural network model are randomly divided according to a ratio of 4:1. The training times of the model are set to 100, the learning rate is set to 0.1, and the minimum error of the training target is 0.001.

#### 2.5.2. Partial LEAST Squares Regression

Partial least squares regression (PLSR) [28] was used to predict the moisture content of *Agaricus bisporus* by analyzing the multivariate statistical regression model between the diffuse reflectance spectral data and the measured moisture content of *Agaricus bisporus*. First, the data were preprocessed, such as by removing outliers and missing values, and samples were divided into a training set and a test set according to a ratio of 4:1. Then, the PLSR model of the training set data was established to obtain the mapping relationship between the spectral data and the moisture content of *Agaricus bisporus*. After that, the test set data are used to evaluate the established PLSR model, and the root mean square error (RMSE) of the model is calculated. Then, according to the model evaluation results, the optimal factor number k is selected, and the model parameters are adjusted to optimize the PLS regression model. Finally, the optimized PLSR model is used for prediction and analysis.

#### 2.5.3. Optimization Algorithm

**Figure 8:**
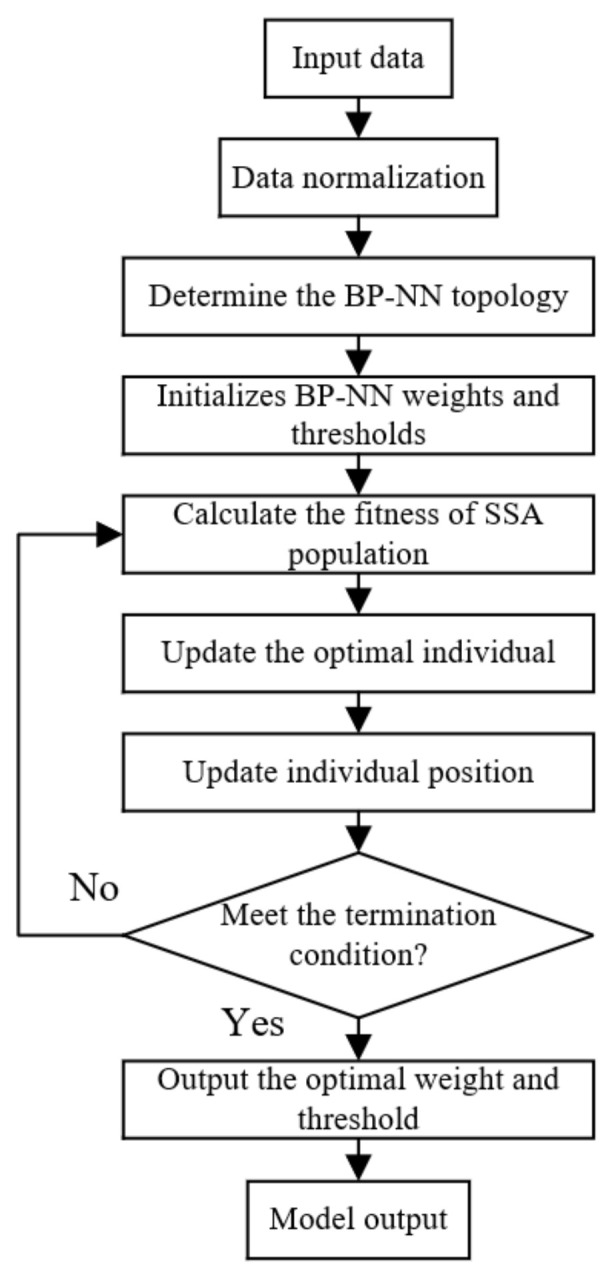
SSA optimizes the BP-NN process.

The Sparrow Search Algorithm (SSA) [29,30] is an optimization algorithm based on the behavior of sparrow behavior of foraging and avoiding predators, and it has strong optimization ability and fast convergence speed. To further improve the prediction performance of the BP neural network model, SSA is selected to optimize the BP neural network. The optimization steps are shown in Figure 8.

### 2.6. Whiteness Detection Method

In this work, the whiteness of *Agaricus bisporus* was detected by spectrophotometry, and the whiteness value of *Agaricus bisporus* was obtained by the CIE Ganz whiteness formula combined with visible spectrum technology and the characteristics of *Agaricus bisporus* samples. The method comprises the following steps: First, the spectral reflectance of the surface of the *Agaricus bisporus* and relative spectral power distribution data of an illuminant were acquired by using a self-developed device, and these data were combined with the spectral tristimulus value (${\bar{x}}_{10}\left(\lambda \right)$, ${\bar{y}}_{10}\left(\lambda \right)$, ${\bar{z}}_{10}\left(\lambda \right)$) of a 10° field of view specified by the CIE (International Commission on Illumination).

Then, the tristimulus values X, Y and Z were calculated according to the equal interval wavelength method recommended by the CIE, and finally, the whiteness value of *Agaricus bisporus* samples was calculated by the CIE Ganz whiteness formula.

To obtain the parameters needed to calculate the Ganz whiteness formula, the tristimulus values were calculated according to the equal-interval wavelength method. The tristimulus values X, Y and Z under the CIE-XYZ color system can be multiplied by the white stimulus function *ψ(λ)* with the CIE spectral tristimulus values and calculated in the range of 450 nm~760 nm. The formula is shown as follows.

(4) $$\left\{ \begin{array}{l} X_{10}=K\sum \delta \left(\lambda \right)R\left(\lambda \right){\bar{x}}_{10}\left(\lambda \right)\Delta \left(\lambda \right)\\ Y_{10}=K\sum \delta \left(\lambda \right)R\left(\lambda \right){\bar{y}}_{10}\left(\lambda \right)\Delta \left(\lambda \right)\\ Z_{10}=K\sum \delta \left(\lambda \right)R\left(\lambda \right){\bar{z}}_{10}\left(\lambda \right)\Delta \left(\lambda \right) \end{array}\right.$$

where $\bar{x}\left(\lambda \right),\bar{y}\left(\lambda \right),\bar{z}\left(\lambda \right)$ refers to the spectral tristimulus value with a field of view of 10° for the standard observer defined by the CIE. *R(λ)* refers to the relative spectral power distribution of the illuminant, *δ(λ)* refers to the measured spectral reflectance of the object under 0/d observing conditions, and the constant *K* is the normalized constant, which can be obtained by Equation (5), where the integral range is 450–760 nm.

(5) $$K=\frac{100}{\int_{450}^{760}\psi \left(\lambda \right){\bar{y}}_{10}\left(\lambda \right)d\lambda }$$

where *ψ(λ)* refers to the white stimulus function: that is, the human eye’s perception of color, which can be obtained by Equation (6).

(6) ψ(λ)=R(λ)δ(λ)

where *δ(λ)* refers to the spectral reflectance of the sample and *R(λ)* refers to the relative spectral power of the illumination source.

According to the CIE Ganz whiteness formula, the above data are substituted into the formula for calculating the whiteness value. The formula is shown in Equation (7).

(7) $$W_{10}=Y_{10}+800\left(x_{n}-x_{10}\right)+1700\left(y_{n}-y_{10}\right)$$

where $\left(x_{n},y_{n}\right)$ refers to the chromaticity coordinates of the standard whiteboard under the standard D65 light source, $Y_{10}$ and $\left(x_{10},y_{10}\right)$ refer to the luminance factor and chromaticity coordinates of the sample under the standard D65 light source, respectively, and the chromaticity coordinate $\left(x_{10},y_{10}\right)$ is represented by Equation (8).

(8) $$\left\{ \begin{array}{l} x_{10}=\frac{X_{10}}{X_{10}+Y_{10}+Z_{10}}\\ y_{10}=\frac{Y_{10}}{X_{10}+Y_{10}+Z_{10}} \end{array}\right.$$

where $X_{10}$, $Y_{10}$ and $Z_{10}$ refer to the tristimulus value under the CIE-XYZ system.

## 3. Results

### 3.1. Calibration and Preprocessing Results

#### 3.1.1. Calibration Results

**Figure 9:**
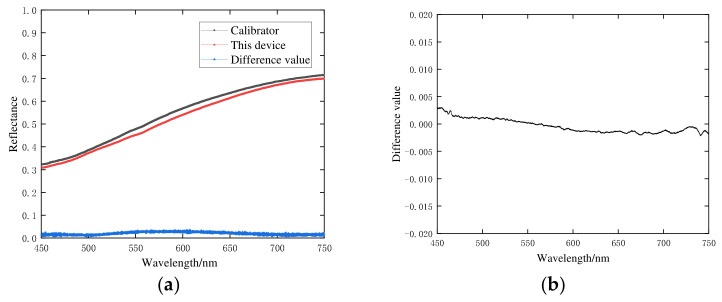
The results of spectral calibration analysis. (**a**) Spectral calibration image; (**b**) Spectral calibration difference curve.

The spectral data from 200 mushrooms were collected using the Ocean spectrometer and the portable spectrometer we constructed, respectively, and each sample was measured three times repeatedly. The results of spectral calibration analysis are shown in Figure 9. The spectral difference curve of the spectral data of the *Agaricus bisporus* sample after calibration is shown in Figure 9a. According to the spectral calibration difference curve Figure 9b, the spectral difference after calibration is less than 0.5%.

#### 3.1.2. Preprocessing Results

To further compare the processing effects of the four kinds of pretreatment methods on the original spectrum, the four kinds of pretreatment spectral data were input into the BP neural network model to establish the water content prediction model of *Agaricus bisporus*. The modeling results are shown in Table 2.

The BP neural network model based on the spectral data processed by MSC has a better effect and a higher correlation coefficient of the training set $R_{C}^{\;2}$ (0.84) and the test set correlation coefficient $R_{p}^{\;2}$ (0.80) and it has a lower RMSEC for the training set (15.01%) and RMCEP for the test set (18.26%).

### 3.2. Dimensionality Reduction Results

#### 3.2.1. SPA Results

The root mean square error (RMSE) is used to determine the relatively best characteristic wavelength selected by the SPA algorithm. Based on the location of the local minimum in the curve between the RMSE and the number of characteristic wavelengths, the optimal number of characteristic wavelengths can be determined. For the moisture content data, when the number of variables is 6, the RMSE is the smallest, approximately 0.0086. Therefore, the number of characteristic wavelengths ultimately selected by SPA is 6, and the selected characteristic wavelengths are 475 nm, 490 nm, 650 nm, 690 nm, 730 nm and 740 nm, respectively. For the whiteness data, when the number of variables is 32, the RMSE is the smallest: approximately 8.93. 

#### 3.2.2. PCA Results

**Figure 10:**
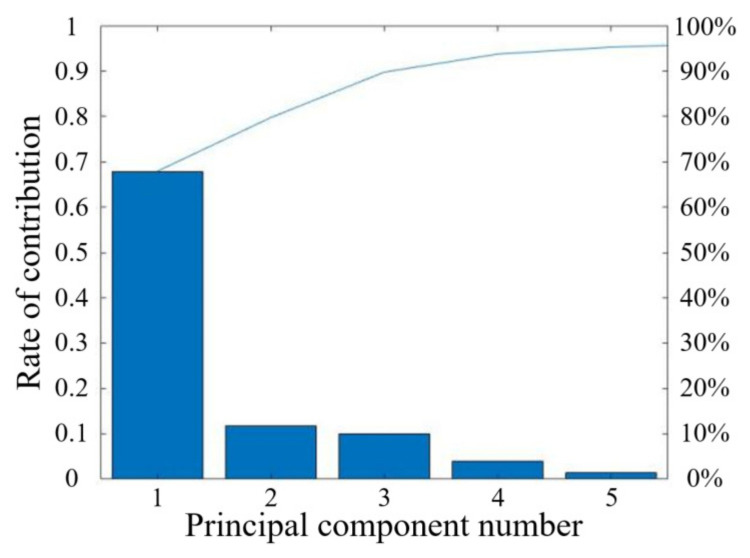
The results of PCA.

The PCA algorithm is used to extract the principal components of the original spectral data for whiteness prediction, and the contribution rate and cumulative contribution rate of each principal component are shown in Figure 10.

It can be seen that in the process of extracting the original data by PCA, the cumulative contribution rate of the first five principal components is more than 95% according to Figure 10, which indicates that the first five principal components can reflect most of the original spectrum, so the first five main components can be selected as the research object.

### 3.3. The Test Results of the Moisture Content

#### 3.3.1. Predicted Results of Moisture Content

To select the best method for predicting the moisture content of *Agaricus bisporus*, a BP neural network prediction model and PLSR prediction model were established by using the visible spectrum data of *Agaricus bisporus* (a total of 200 samples) and the standard value of the moisture content of *Agaricus bisporus*, respectively. The samples were randomly divided into a training set and a test set according to the ratio of 4:1, the training set was used to establish the model, and the test set was used to verify the availability of the model. Using the training set correlation coefficient ($R_{C}^{\;2}$) and test set correlation coefficient ($R_{p}^{\;2}$), RMSEC and RMSEP were used to evaluate the accuracy of the model. The modeling results of different models are shown in Table 3.

According to the modeling results of Table 3, the performance index of the BP model is higher than that of the PLSR model and the BP network model based on the whole spectrum of *Agaricus bisporus*. Compared with the PCA algorithm, the SPA has a higher accuracy of the model built by the selected characteristic wavelengths. It can be seen that the BP neural network model established by using the characteristic wavelength selected by SPA is the best, where $R_{p}^{\;2}$ and *RMSEP* are 0.91 and 8.07%, respectively, which have higher prediction accuracy compared with other models.

#### 3.3.2. The Result of Optimization

**Figure 11:**
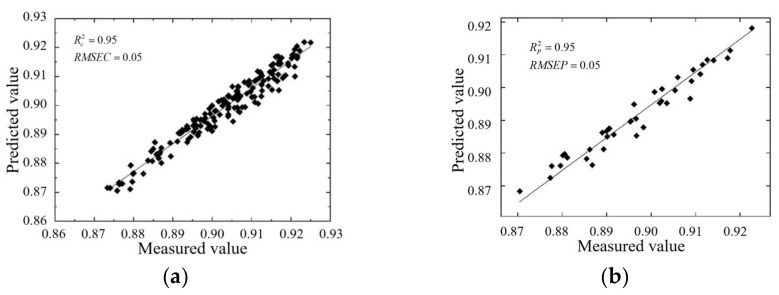
The test result of the SPA-SA-BP model. (**a**) The training set results; (**b**) The test set results.

To further improve the prediction performance of the BP neural network model, SSA is selected to optimize the BP neural network model. First, the parameters of the SPA-SSA-BP prediction model are set: the sparrow evolution population number is 10, the sparrow evolution time is 20, the proportion of discoverers is 70%, the proportion of scouts is 20%, the alert value is 60%, the BP neural network learning rate is 0.1, the training time is 1000, and the minimum error of the training target is 0.001. The test results of the network model optimized by SSA are shown in Figure 11.

The test results of the SPA-SSA-BP model and SPA-BP model are shown in Table 4.

It can be seen that the training set $R_{C}^{\;2}$ and *RMSEC* of the SPA-SSA-BP model are 0.95 and 5.11%, respectively, and the prediction set $R_{p}^{\;2}$ and *RMSEP* are 0.95 and 5.04%, respectively, according to Table 4. Compared with the test results of the SPA-BP neural network model, the training set accuracy is improved by 3%, the root mean square error of training is reduced by 2.12%, the prediction set accuracy is improved by 4%, and the root mean square error of testing is reduced by 3.03%. The prediction accuracy of the BP neural network model optimized by SSA has been significantly improved.

### 3.4. The Results of Whiteness Test

#### 3.4.1. Correlation Analysis of the Whiteness of *Agaricus bisporus*

**Figure 12:**
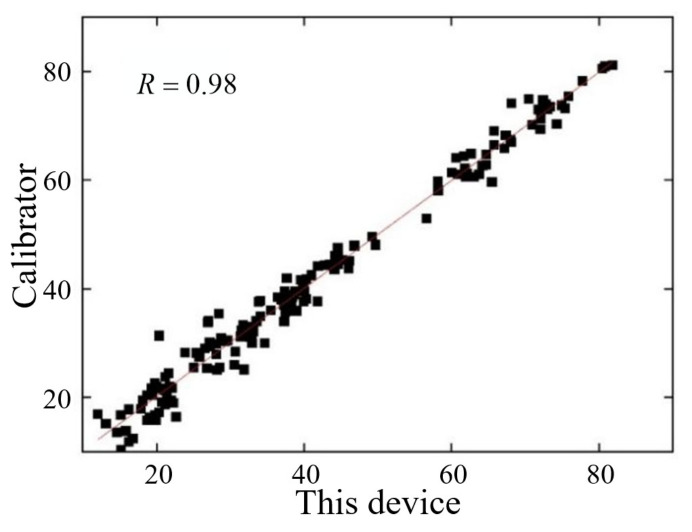
The results of correlation analysis.

In this work, spectral colorimetry was used to measure the whiteness of four grades of *Agaricus bisporus*, and linear regression analysis was carried out on the whiteness data measured by the self-developed device and the Ocean spectrometer. The results of the correlation analysis between the two are shown in Figure 12. There is a significant correlation between the results of whiteness measured by the spectrometer and the results measured by this device. The Pearson correlation coefficient was 0.98, which indicated that the device had a certain reliability for the measurement of the whiteness of *Agaricus bisporus*.

#### 3.4.2. Whiteness Grading Results of *Agaricus bisporus*

**Figure 13:**
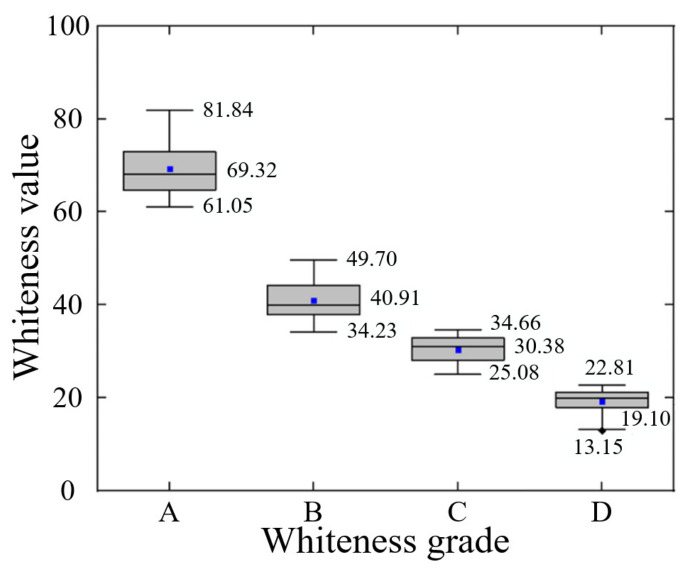
Whiteness Grading Results of *Agaricus bisporus*.

The spectrum acquisition device in this paper is used to measure the whiteness value of each grade of *Agaricus bisporus*, and the statistical data are shown in Figure 13. The ranges of whiteness were [61.05, 81.84], [34.23, 49.7], [25.08, 34.66] and [13.15, 22.81], and the medians were 69.32, 40.91, 30.38 and 19.1, respectively. There were significant differences among the data of each group (*p* < 0.01). The results showed that the device had a certain reliability for the classification of different whiteness grades of *Agaricus bisporus*.

#### 3.4.3. The result of the whiteness prediction model

**Figure 14:**
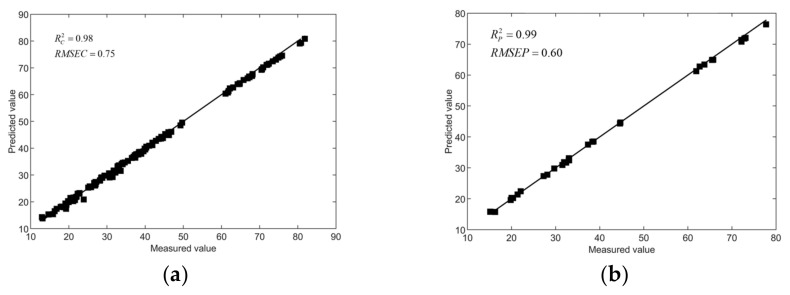
Prediction results of whiteness value of *Agaricus bisporus*. (**a**) The training set results; (**b**) The test set results.

Whiteness is one of the important indicators of the freshness of *Agaricus bisporus*. In the process of picking, storage and sales, *Agaricus bisporus* will produce browning due to bumping and long storage times. Predicting the whiteness of *Agaricus bisporus* shows that the device has the ability of model regression analysis, which can provide a certain basis for judging the freshness of *Agaricus bisporus*. Therefore, 160 samples were collected in this work, including 128 samples in the training set and 32 samples in the prediction set. The whiteness prediction model was established by using a BP neural network, and the prediction results are shown in Figure 14, where the results for the prediction set $R_{p}^{\;2}$ and *RMSEP* are 0.99 and 0.60 for the test dataset, respectively.

## 4. Discussion

### 4.1. Comparison of Related Studies

To further explore the feasibility of this study, the research results of several scholars on the moisture content and whiteness of *Agaricus bisporus* were summarized and compared, and the results are shown in Table 5.

In terms of moisture content prediction, the accuracy of the model established in this work is significantly higher than that of other studies, and the RMSE of the test results is relatively lower according to Table 5. There are few studies on the whiteness detection of *Agaricus bisporus*. Compared with Zhao et al. [33], this work can be more portable to detect the whiteness of *Agaricus bisporus*. More importantly, the device in this work can detect the two parameters of moisture content and whiteness at the same time, which is more accurate for the quality detection of *Agaricus bisporus* and which is not available in other studies.

### 4.2. Error Analysis

According to the experimental results, although the detection results of moisture content and whiteness of the portable *Agaricus bisporus* spectrum acquisition device built in this work have been significantly improved compared with those of other studies, there are still some errors. The main reasons are analyzed as follows:

(1)  The spectral calibration of the device is based on the Ocean spectrometer, and the Ocean spectrometer itself has errors, so the research device will inevitably have prediction errors.
(2)  To reduce the complexity of feature extraction, not all features are used as input variables, and some features are discarded, which will produce prediction errors.
(3)  The limited number of samples leads to errors caused by the inaccuracy of the model training results.
(4)  Random errors caused by unprofessional operation in the process of experimental operation.
(5)  This device uses LED lamp beads instead of a D65 standard light source, so a measurement error will be generated.

The whiteness and moisture content are important indicators of the freshness of *Agaricus bisporus* and can determine the quality of *Agaricus bisporus*. To further reduce the measurement error of the whiteness value of the moisture content, the experimental operation can be optimized in the future, and the spectral characteristics can be selected as much as possible on the premise of ensuring that the prediction accuracy is not reduced, or a combined light source or a filter can be added to the existing light source to more accurately simulate the D65 standard light source, thereby improving the accuracy of the moisture content and whiteness measurement.

## 5. Conclusions

In this work, a portable spectrum acquisition device for *Agaricus bisporus* was built, and a light source module, a spectrum acquisition module and a spectrum processing unit were designed. The correlation coefficient of the diffuse reflectance spectral data of *Agaricus bisporus* samples collected by this device and the Ocean Spectromeper is 0.98, which verifies the reliability of the results measured by this device. The prediction models of moisture content and whiteness based on the BP neural network and PLSR were constructed, the spectral characteristic wavelengths of *Agaricus bisporus* were extracted by SPA and PCA algorithms, and the parameters of the BP neural network model were optimized by the SSA algorithm. The R^2^ of the final predicted values of moisture content and whiteness were 0.95 and 0.99, the RMSEPs were 5.04% and 0.60, respectively, and there were significant differences among the whiteness grades (*p* < 0.01). The results show that the portable spectrum acquisition and analysis device can be used for the accurate and rapid detection of the quality of *Agaricus bisporus*.

## Figures and Tables

**Table 1:** Manual grading standard for whiteness.

Whiteness Grade	Characteristic
Level 1	Pure white, no brown spots
Level 2	White with slight brown spots
Level 3	Milky white with brown spots
Level 4	Yellowish brown with numerous brown spots

**Table 2:** Comparison of processing results of different models.

Modeling Method	Training Set		Prediction Set	
	$R_{C}^{\;2}$	RMSEC of Moisture Content (%)	$R_{p}^{\;2}$	RMSEP of Moisture Content (%)
SG-BP	0.83	15.38%	0.78	21.42%
SNV-BP	0.83	16.12%	0.79	19.21%
MSC-BP	0.84	15.01%	0.80	18.26%
Normalization-BP	0.81	18.13%	0.78	20.35%

**Table 3:** Experimental results of different models.

Modeling Method	Training Set		Prediction Set	
	$R_{C}^{\;2}$	RMSEC (%)	$R_{p}^{\;2}$	RMSEP (%)
Full-PLSR	0.78	18.21%	0.76	22.15%
Full-BP	0.83	15.06%	0.80	19.24%
SPA-BP	0.92	7.23%	0.91	8.07%
PCA-BP (5 components)	0.89	9.69%	0.91	8.37%
PCA-BP (4 components)	0.90	8.74%	0.90	8.62%
PCA-BP (3 components)	0.90	8.70%	0.90	9.38%
SPA-PLSR	0.90	10.34%	0.86	13.45%
PCA-PLSR	0.87	11.26%	0.87	12.17%

**Table 4:** Comparison of processing results of the SPA-SSA-BP and SPA-BP models.

Modeling Method	Training Set		Test Set	
	$R_{C}^{\;2}$	RMSEC (%)	$R_{p}^{\;2}$	RMSEP (%)
SPA-BP	0.92	7.23%	0.91	8.07%
SPA-SSA-BP	0.95	5.11%	0.95	5.04%
Δ	3%	−2.12%	4%	−3.03%

**Table 5:** The results of this work and other scholars.

Detection Technology	Detect Content	Model	R^2^	RMSEP	Portable/Fixed Detection Device	Ref.
			Significant Difference (*p*)			
Visible-near infrared spectroscopy	Moisture content	SSA-SPA-BP	0.95	5.11%	Portable	This work
	Whiteness	SPA-BP	0.99	0.60		
			*p* < 0.01			
Near-infrared hyperspectral imaging	Moisture content	PLSR	0.96	6.31%	Fixed	[12]
Visible-near infrared spectroscopy	Moisture content	PLSR	0.78	8.81%	Portable	[31]
Hyperspectral imaging	Moisture content	PCR	0.75	0.74%	Fixed	[32]
Machine vision and image processing	Whiteness	CIE-Ganz whiteness formula	*p* < 0.01		Fixed	[33]

## Data Availability

The data presented in this study are available upon request from the corresponding author. The data are not publicly available.
